# Whole-exome sequencing of *de novo* genetic variants in a Chinese family with a sporadic case of congenital nonsyndromic hearing loss

**DOI:** 10.12688/f1000research.27739.2

**Published:** 2021-08-24

**Authors:** Sijing Hu, Hao Zhang, Yunqiang Liu, Mohan Liu, Jingjing Li, Shunyao Liao

**Affiliations:** 1 Diabetes Center & Institute of Organ Transplantation, Sichuan Provincial People’s Hospital, University of Electronic Science and Technology of China, Chengdu, China, 610072, China; 2 Department of Medical Genetics and Division of Morbid Genomics, State Key Laboratory of Biotherapy, West China Hospital,, West China Medical School, Sichuan University,, Chengdu, China, 610041, China

**Keywords:** Whole-exome sequencing, de novo pathogenic variant, nonsyndromic hearing loss, compound heterozygosity, genetic and environmental interaction

## Abstract

**Background:** We examined the genetic variants of a Chinese family with a 22-month-old infant with sporadic non-syndromic sensorineural hearing loss (NSHL).

**Methods: **The whole-exome sequence data in the family, especially the *de novo* variants presented in the patient, were analyzed and the effect of the disease-causing genetic variants on the protein expression level and cellular localization were examined by cell-based functional assay.

**Results:** The infant had no known NSHL-causing variants, except two compound heterozygous variants in connexin26 gene *GJB2*; one was the c.79G>A, c.341A>G haplotype from the asymptomatic mother who was benign, and the other was a *de novo* pathogenic c.262G>C (p.A88P). *In vitro*, *GJB2 *with c.262G>C was weakly expressed and displayed a punctate distribution in the cytoplasm and cytomembrane, while wild type *GJB2* was robustly expressed in the cytomembrane. We deduced that the *de novo* pathogenic *GJB2* c.262G>C exacerbated loss-of-function in the context of leaky variants c.79G>A, c.341A>G in the patient. Interestingly, further analysis of exome sequences revealed that the occurrence of *de novo *pathogenic variants in the infant was frequent. Among the total~47,000 variants, 143 were *de novo* in the patient, whereas among all 74 variants predicted to be pathogenic/likely pathogenic, 21 were heterozygous and two were homozygous *de novo*. The occurrence rate of *de novo* deleterious variants was much higher (31.1%, 23/74) than that in total (0.34%, 143/47,000). It is notable that most genes with *de novo* deleterious variants were environment-sensitive, such as *GJB2*, *MNK1*, *MNK2, MUC4*, *RAD21 *and DNA copy number variations.

**Conclusions: **The full picture of genetic variants in the exome might help us to interpret the NSHL-causing variants. More research is needed into the causes of *de novo* deleterious variants and gene-environment interactions in congenital NSHL.

## Introduction

Hearing loss is one of the most common birth defects. The pathogenic variants of non-syndromic sensorineural hearing loss (NSHL) (OMIM: 121011) were found in 49 genes (Cite https://www.ncbi.nlm.nih.gov/books/NBK1272/). Variants in the Gap Junction Protein Beta 2 gene ( *GJB2*, HGNC: 4284), which encodes a beta-2 gap junction protein (connexin 26; Cx26), have been shown to be the leading genetic cause of NSHL. *GJB2* *-*related autosomal recessive deafness explains approximately 50% of congenital autosomal recessive deafness, and *GJB2* *-*related autosomal dominant deafness is extremely rare.

GJB2 constitutes cell-to-cell channels and facilitates the intercellular exchange of ions and molecules. ^1^ The amino acid alanine at position 88 (p.A88) of GJB2, which is located in the second transmembrane domain of Cx26, is highly conserved in vertebrates. To date, five studies have reported five nucleotide changes in the p.A88 coding region that resulted in distinct clinical abnormalities and different inheritance patterns. Frei *et al*. first reported the heterozygous c.262G>T (p.A88S) variant in a male Austrian patient with NSHL. As the proband’s mother was an asymptomatic carrier, the authors inferred that the missense variant could be connected to deafness but not in a simple and monogenetic disease model. ^2^ Gravian *et al*. found that the c.262G>C (p.A88P) variant in compound heterozygosity with the nonpathogenic variant p.V27I in an Argentina child with profound deafness, implicating the destructive potential of the c.262G>T variant. ^3^ Other researchers have reported 3 patients with p.A88 coding variants at the 263rd nucleotide: one case was the c.263C>G (p.A88G) variant in a Tunisian girl with autosomal recessive NSHL, where her consanguineous parents were healthy carriers ^4^; another case was the c.263C>A (p.A88E) variant in a Chinese patient with sporadic NSHL where the variant was in compound heterozygosity with the disease-causing c.235delC ^5^; and another case was the c.263C>T (p.A88V) variant in a Japanese girl with severe keratitis-ichthyosis-deafness syndrome and septic complications, with unaffected parents. ^6^ To date, by directly sequencing the *GJB2* genetic region, studies have demonstrated that variants in GJB2 p.A88 have been associated with hearing loss in children. However, descriptions of the penetrance of the variants have been inconsistent.

On the other hand, the *GJB2* c.79G>A (p.V27I, rs2274084) *in cis* with c.341A>G (p.E114G, rs2274083) forming a haplotype of p.[V27I; E114G] occurs frequently in East Asian populations. ^7^^,^^8^ P.V27I is located in the first transmembrane domain and p.E114G is located in the intracellular loop of Cx26.Both are classified as benign polymorphisms. However, several clinical studies have found that the p.[V27I; E114G] haplotype is a risk factor for hearing impairment, ^7^^-^^10^ and functional assays *in vitro* have demonstrated that the channel activities of VG (p.E114G variant only) and IG (both p.V27I; p.E114G variants) were reduced. ^11^ However, as both genotypes were detected in both patients and controls, ^7^^-^^10^ the exact pathogenic role of these variants in NSHL remains controversial.

Whole-exome sequencing enables a comprehensive and precise genetic investigation of congenital disorders and allows us to search highly heterogeneous genetic causes. This study aimed to explore possible molecular abnormalities in a Chinese non-consanguineous family with a 22-month old daughter suffering from NSHL. We carried out whole-exome sequencing, assessed the cytological/clinical characteristics of the genetic variants, specifically in GJB2 genetic variants, and evaluated the possible cause of *de novo* pathogenic variants in the patient’s exome.

## Methods

### Patient details

The family included in this study is of Han Chinese heritage and resides in Chengdu City of Southwest China. The proband was a 22-month-old girl with NSHL who had previously been born in our hospital by spontaneous delivery at full term. Both of her parents were healthy during pregnancy. The baby failed the newborn hearing examination but no prenatal or postnatal risk factors for hearing loss were identified. Similarly, no family history of hearing abnormalities was reported. When the parents brought the 22-month-old child back to the hospital in October 2018, physical, biochemical, and otoscopic examinations were carried out. A CT scan of the temporal bones and MR analysis of the child’s head were also done to search for any organic brain lesions, and pure tone audiometry was performed in the girl and her parents.

Written informed consent was obtained from both parents for them and their daughter to participate in the study. The work was approved by the Research Ethics Committee of Sichuan Provincial People’s Hospital, School of Medicine, University of Electronic Science and Technology of China.

### Whole-exome and mitochondrial DNA sequencing

Blood genomic DNA and mitochondrial DNA were extracted from all family members according to standard procedures (Abcam, Cambridge, UK) and stored in -20°C. The DNA concentration and quality were examined using a NanoDrop 2000 (Thermo, USA).

The whole-exome sequencing, the entire mitochondrial DNA and genetic variations analysis are described in our previous work. ^12^ The fragmented genomic DNA was enriched using a NimbleGen probe capture array SeqCap EZ Exome Kit v3.0 (Roche NimbleGen, Inc. Madison, WI). The kit using the SeqCap advanced design algorithm coupled with 2.1 million long oligonucleotide probes to achieve superior target enrichment performance, and detect genetic variants with ~98% sensitivity and 99% specificity. The enriched DNA fragments passed the qPCR test, and the size distribution and concentration of these DNA fragments were examined using the Agilent Bioanalyzer 2100 (Agilent Technologies, Santa Clara, CA). The samples were sequenced on an Illumina NovaSeq 6000 (Illumina, San Diego, CA), and two parallel reactions were performed. Raw image files were processed by the BclToFastq (Illumina) for base calling to generate the raw data. The low-quality variations were filtered out using the quality score = 20 (Q20). The sequencing reads were aligned to the NCBI human reference genome (hg19) using Burrows-Wheeler Aligner (version 0.6.2). SAMtools and Pindel were used to analyze single nucleotide polymorphisms (SNPs) and insertion/deletion of the sequence. The coding variants and CNVs were filtered out in the dbSNP135, Exome Variant Server, 1000 Genomes, and in-house database with more than 100,000 Chinese exomes (Joy Oriental Co. Beijing, China). The variants and CNVs were also searched in the Human Gene Mutation Database (HGMD), ClinVar, and the Online Mendelian Inheritance in Man database (OMIM).

The entire mitochondrial DNA was enriched by long-range PCR followed by massively parallel sequencing.

The related primers are listed in Table 1.

**Table 1.** Primer list.

Primer Name	Primer Sequence (5′ to 3′)
***Primers for amplifying genomic DNA***	
***GJB2*-F**	5′-AGCAAACCGCCCAGAGTAGAAG-3′
***GJB2*-R**	5′-AAGATGACCCGGAAGAAGATGCT-3′
***Primers for HA-tagged protein expression vector construction***	
**WT-F**	5′-CTTGGTACCGAGCTCGGATCCATGGATTGGGGCACGCTG-3′
**WT-R**	5′-TGCTGGATATCTGCAGAATTCAACTGGGCAATGCGTTAAACTG-3′
**Mut-F**	5′-CCGCTCCTAGTGGCCATGCACGTGG-3′
**Mut-R**	5′-GTGGCGTGGACACGAAGATCAGCTGCA-3′
***Mitochondrial genome DNA sequencing***	
**mt16426F**	5′-CCGCACAAGAGTGCTACTCTCCTC-3′
**mt16425R**	5′-GATATTGATTTCACGGAGGATGGTG-3′

### Sanger sequencing

Sanger sequencing was used to verify the variations of the candidate genes in the family members. ^12^ The primers for amplifying the targeted region of candidate genes are also shown in Table 1.

### Variant functional assay

The wild-type *GJB2* cDNA and *GJB2* cDNA with the c.262G>C variant were amplified with the primers shown in Table 1. The HA-tagged wild-type and mutant coding sequences were inserted into the pcDNA3.1(+) vector (Invitrogen, Carlsbad, CA) using the Mut Express® II Fast Mutagenesis kit V2 (Vazyme, Nanjing, China). Human H1299 cells (ATCC, Manassas, VA) were transfected to express the vectors using the jetPRIME Transfection Kit (Polyplus, Illkirch, France) according to the manufacturer’s instructions. After 48 hours of transfection, the cells were collected for immunoblotting and immunohistochemical analysis.

### Protein analysis

The online Clustal Omega and Conseq software programs were used to align the amino acid sequences in a variety of species. The Polyphen-2, SIFT and MutationTaster programs were used to predict the variants as “damaging” or “possibly damaging”. The clinical interpretation of genetic variants by the American College of Medical Genetics and Genomics/Association for Molecular Pathology (ACMG/AMP) guidelines was followed to classify the variants into “benign”, “likely benign”, “uncertain significance”, “likely pathogenic”, and “pathogenic”. ^13^

### Immunoblotting and western blot analysis

For immunoblotting analysis, the cells were lysed with RIPA buffer containing protease inhibitor cocktail (Roche Diagnostics GmgH, Mannheim, Germany). The lysate was centrifuged, collected, and boiled in SDS loading buffer. Then, the proteins were separated on 10% SDS-polyacrylamide gels. After the proteins were transferred onto polyvinylidene difluoride membranes (Millipore, USA), the membranes were blocked and incubated with rabbit polyconal anti-HA antibody (Dilution: 1:1000, Cat No.: 51064-2-AP, Proteintech, Chicago, USA) and the secondary antibodies (Dilution: 1:10000, Cat No.: BA1055, Boster Wuhan, China), and the protein bands were visualized using an HRP chemiluminescent substrate kit (Millipore) and a ChemiDoc XRS+ System (Bio-Rad Company, Berkeley, CA).

For immunohistochemical analysis, the cells were fixed with 4% paraformaldehyde in 0.1 M PBS for 20 min and then rinsed three times in PBS. Then, the coverslips were immersed in cold methanol for 15 min at -20°C. The primary antisera and dilutions were as follows: rabbit anti-HA antibody at 1:100 (Proteintech) for WT/MUT GJB2. After incubation with primary antiserum at 4°C overnight, the cells were rinsed in PBS three times before adding Alexa Fluor 488- and/or Alexa Fluor 594-conjugated secondary antibodies (Dilution: 1:500, Cat. No.: A-11008, Invitrogen). ER was stained with ER-Tracker Red at 1:2000 dilutions (Beyotime, Shanghai, China) for 10 min at room temperature. Preimmune rabbit serum was used as the primary antibody for the negative controls. The images were visualized using a Zeiss Axio Imager Z2 microscope (Carl Zeiss, Jena, Germany).

## Results

### Clinical characteristics of the patient’s family

**Figure 1.**
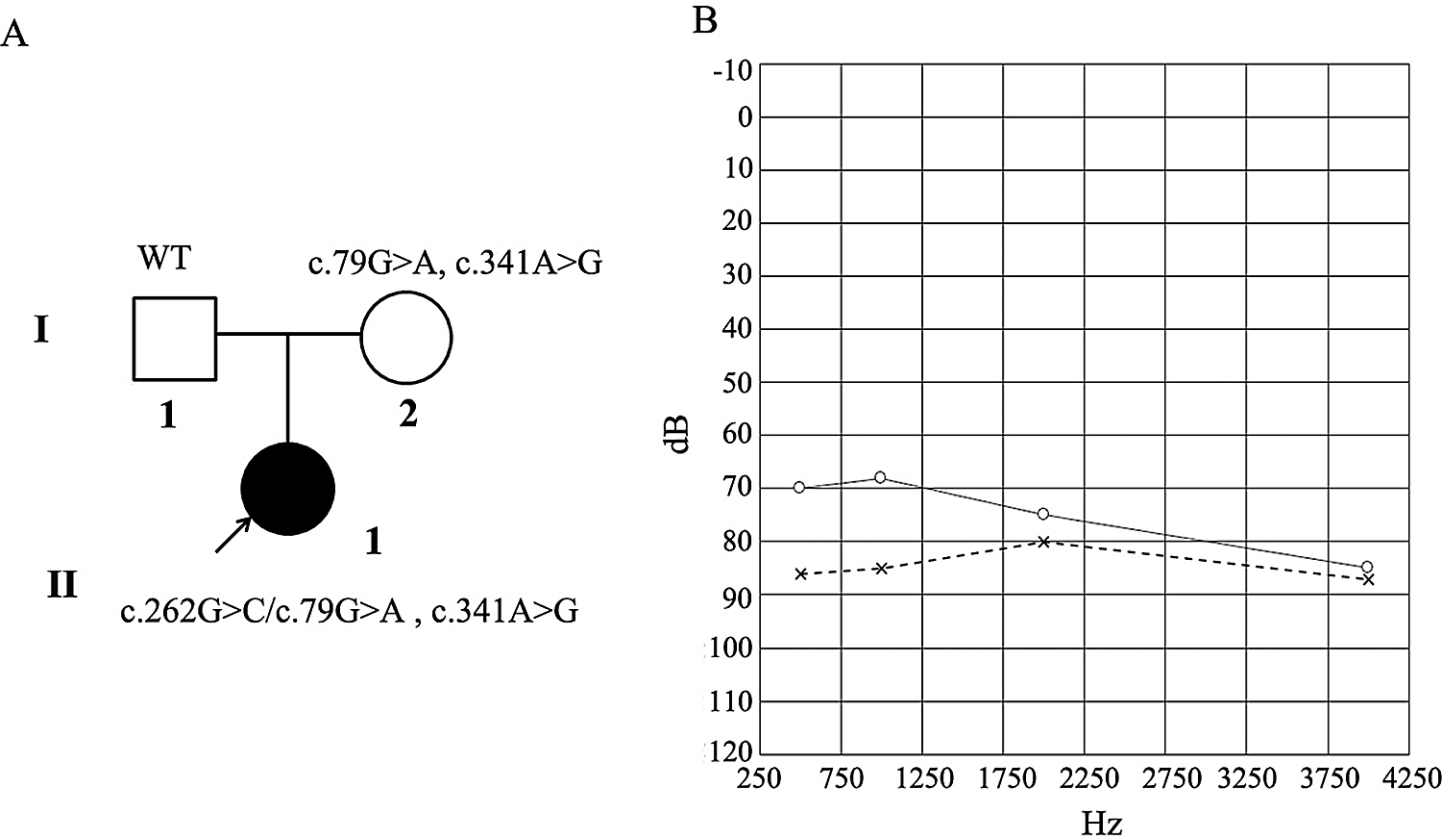
Genetic characteristics of the family, the pedigree of the family (A) and audiograms for the proband (B). The horizontal axis of the audiogram shows the tone frequency (Hz) and the vertical axis displays hearing level (dBHL). Severe hearing loss was classified as a pure-tone average between 70-95 dBHL. х, left ear, о, right ear.

The pedigree of the family is shown in Figure 1A. The kinship connection between the proband and parents is confirmed by the exome sequence data. ^21^ The proband had normal physical, biochemical and otoscopic evaluations. No abnormality was found in her cranium by MR examination or in her cochlear, vestibular, and semicircular canals by CT scan. Pure tone audiometry indicated that her left and right hearing thresholds were 78 dB and 87 dB, respectively, with severe hearing loss in both ears ( Figure 1B). Since there was no family history of HL and the child’s parents had normal hearing, the affected infant is considered to be a sporadic case of NSHL.

### Analyses of variants detected in GJB2 gene in the patient’s exome

**Figure 2.**
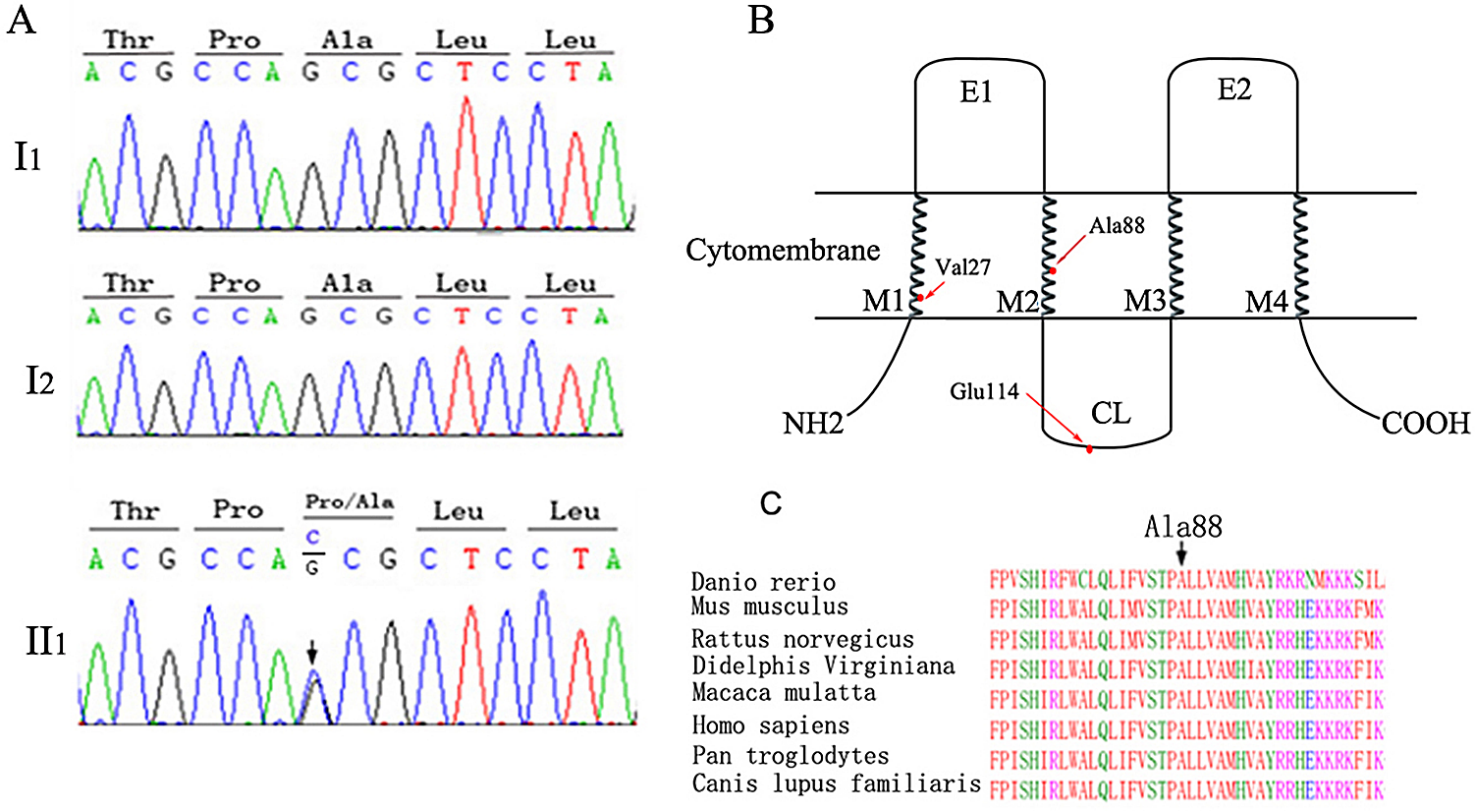
DNA and protein sequence analysis of GJB2. (A) The DNA sequence electropherograms (I1 father, I2 mother, II1 daughter) revealing wild-type sequence of the parents and *de novo* 262G to C transversion from their daughter (black arrow). (B) The schematic diagram of Cx26, where M1-M4 are transmembrane domains, E1-E2 are two extracellular loops, CL is intracellular loop, and NH2 and COOH is N- and C-cytoplasmatic termini respectively. The non-pathogenic c.79G>A (p.V27I), c.341A>G (p.E114G) is in both the M1 and intracellular loop. The c.262G>C (p. A88E) is in the M2 of Cx26. (C) The alignment of the Cx26 amino acid sequences among the different species. The alanine at codon 88 is highly conserved.

**Figure 3.**
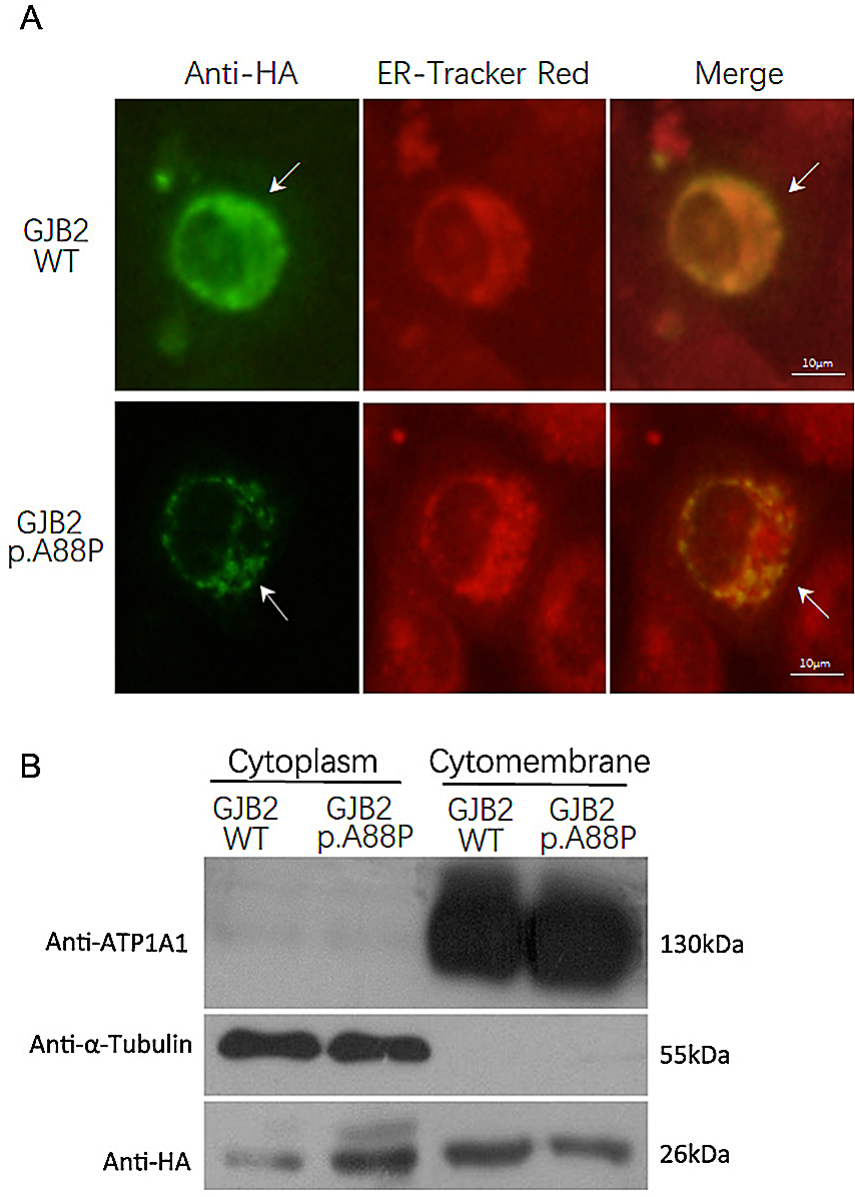
Expression of the p.A88P Cx26 mutants in cells. The immunofluorescence staining showed the wild-type GJB2 was expressed robustly and distributed mainly in the cytomembrane, while the p.A88P mutant was expressed weakly and displayed a punctate distribution in the cytoplasm and cytomembrane (A, arrow); the ER tracker red demonstrated the cytoplasm (A). The immunoblotting confirmed the wild-type GJB2 largely localized in the cytomembrane, while the p.A88P mutants co-localized in the cytoplasm and cytomembrane (B).

The mitochondrial sequencing showed no NSHL-causing variants or large deletions. The exome sequences revealed no known NSHL-causing variants in the family except that the proband had a *de novo* heterogeneous variant c.262G>C in the *GJB2* gene (MAF unknown, Table 2 and Figure 2), whereas her parents were wild type. The c.262G>C variant led to a missense variant of p.A88P, which was graded to be “damaging” with a SIFT score of 0.00 and a Polyphen-2 score of 1.00. To examine the effect of the c.262G>C variant on the protein expression level and cellular localization, we transiently transfected the *GJB2* c.262G>C mutant into H1299 cells and found that the mutant was expressed weakly and displayed a punctate distribution in the cytoplasm and cytomembrane. In contrast, wild-type *GJB2* was expressed robustly and was distributed mainly in the cytomembrane ( Figures 3A & B). This result confirmed that the GJB2 p.A88P mutant may fail to locate into the cell membrane and subsequently reduce the formation of gap junctions in quantity.

**Table 2.** All *de novo* variants which were predicted to be pathogenic or likely pathogenic in the proband’s whole exome.

Gene	Gene anotation	Genetic Location	rs	Proband	Father	Mather	*de novo* variant	Amino acid variation	Type	MAF (in-house database of Joy Oriental Co.)	Clinical significance defined by ACMG
*GJB2*	Gap junction protein beta 2	13q11-q12 (20763459)		Heterozygote	Wild type	Wild type	c.262(exon2)G>C	p.A88P	Missense		Pathogenic
LOC100509263		chr9 (35183448-35183484)	rs1267269489	Homozygote	Wild type	Wild type	c.250(exon1)_c.286(exon1)delCCTCACCTCCCAGGCAGGGCGGCCGGGCAGAGGCGCT	p.P84Pfs*18	Frame shifting	0.007	Pathogenic
*MUC4*	Mucin 4, cell surface associated	3q29 (195506650)		Homozygote	Wild type	Wild type	c.11801(exon2)C>T	p.A3934V	Missense	0.016	Likely pathogenic
		3q29 (195506674-195506675)	rs771640527	Heterozygote	Wild type	Wild type	c.11776(exon2)_c.11777(exon2)delGT	p.V3926Ffs*23	Frame shifting	0.001	Pathogenic
		3q29 (195506677-195506678)		Heterozygote	Wild type	Wild type	c.11773(exon2)_c.11774(exon2)insC	p.D3925Afs*25	Frame shifting	0.001	Pathogenic
		3q29 (195506678-195506679)		Heterozygote	Wild type	Wild type	c.11772(exon2)_c.11773(exon2)insA	p.D3925Rfs*25	Frame shifting	0.000732	Pathogenic
*PHGR1*	Proline, histidine and glycine rich 1	15q15.1 (40648396-40648398)	rs755350345	Heterozygote	Wild type	Wild type	c.141(exon4)_c.143(exon4)delTGG	p.P47_G48delinsP	Inframe deletion	0.000123	Likely pathogenic
*AGAP4*	ArfGAP with GTPase domain, ankyrin repeat and PH domain 4	10q11.22 (46322037)	rs879947525	Heterozygote	Wild type	Wild type	c.1318(exon7)A>G	p.K440E	Missense	0.000259	Likely pathogenic
*MAPK8IP2*	Mitogen-activated protein kinase 8 interacting protein 2	22q13.33 (51043846)		Heterozygote	Wild type	Wild type	c.1816(exon6)G>A	p.E606K	Missense		Likely pathogenic
*ADAMTSL4*	Thrombospondin repeat-containing protein 1	1q21.3 (150529350)		Heterozygote	Wild type	Wild type	c.1750-65(IVS10)A>G		Non-coding		Likely pathogenic
*MAPK8IP1*	Mitogen-activated protein kinase 8 interacting protein 1	11p11.2 (45927951)		Heterozygote	Wild type	Wild type	c.*679(exon12)C>T		Non-coding	0.0000456	Likely pathogenic
*FRG1*	Facioscapulohumeral muscular dystrophy region gene-1	4q35 (190880984)	rs62345304	Heterozygote	Wild type	Wild type	c.538-919(IVS6)T>G		Non-coding	0.012	Likely pathogenic
*ZNF880*	Zinc finger protein LOC400713	19q13.41 (52888074-52888075)		Heterozygote	Wild type	Wild type	c.1241(exon4)_c.1242(exon4)insATCATGAGGTCAGGAGATCGAGACCATCCTGGCTAACAAGGTGAAACC	p.G414delinsGSX,162	Stop gain	0.006	Pathogenic
*RAD21*	protein involved in DNA double-strand break repair, sister chromatid cohesion 1	8q24 (117864951-117864952)		Heterozygote	Wild type	Wild type	c.1162-5(IVS9)_c.1162-4(IVS9)insG		Splice-site		Likely pathogenic
		8q24 (117864952)	rs1419526108	Heterozygote	Wild type	Wild type	c.1162-5(IVS9)A>T		Splice-site		Likely pathogenic
*OPN1MW2*	Opsin 1, medium wave sensitive 2	chrX (153485284-153498649)		Loss of heterozygosity	Wild type	Wild type	loss1(EXON:1-6)(all)		Exon deletion	0.047	Pathogenic
*ARHGEF5*	Rho guanine nucleotide exchange factor 5	chr7 (144059762-144072768)		Loss of heterozygosity	Wild type	Wild type	loss1(EXON:2-12)		Exon deletion	0.05	Pathogenic
chr3		chr3 (15621416-15631119)		Loss of heterozygosity	Wild type	Wild type	loss1		Large CNV deletion	0.0064	Pathogenic
chr10		chr10 (48218794-48237210)		Loss of heterozygosity	Wild type	Wild type	loss1		Large CNV deletion	0.025	Pathogenic
chr19		chr19 (7981506-7985434)		Loss of heterozygosity	Wild type	Wild type	loss1		Large CNV deletion	0.0053	Pathogenic
chr22		chr22 (50320902-50355459)		Loss of heterozygosity	Wild type	Wild type	loss1		Large CNV deletion	0.019	Pathogenic
chr16		chr16 (28723007-28726011)		Single repeat	Wild type	Wild type	gain1		Large CNV duplication	0.039	Pathogenic
*LOC81691*		chr16 (20838379-20839859)		Single repeat	Wild type	Wild type	gain1(EXON:9-11)		Exon duplication	0.0056	Pathogenic

Because heterozygous c.262G>C missense variants were previously found in both patients and healthy carriers in the clinic, ^2^^,^^4^ we rechecked the exome sequences of the family to search for any other possible genetic causes of NSHL. We failed to find any other NSHL-causing variants, but noticed that the mother was a heterozygote of *GJB2* c.79G>A (p.V27I), c.341A>G (p.E114G), the father was wild type, and the affected infant was a heterozygote of c.79G>A (p.V27I) and c.341A>G (p.E114G). As mentioned before, although no significant loss of function has been detected when VG and IG gap junctions coexist with the VE and IE types, the VG and IG types have displayed a moderate deficit in biochemical coupling and reduced channel activity *in vitro*. ^11^ Hence, we deduced that the *de novo* p.A88P mutants in the infant dislocated from the cell membrane, exacerbating GJB2 loss-of-function in the context of the p.[V27I; E114G], whereas the wild-type p.A88 in her mother could compensate for the loss, thus the infant’s compound heterozygosity at p.A88P and p.[V27I; E114G] was affected while her mother is an asymptomatic carrier of p.[V27I; E114G]. This result indicates that the multiple genetic variants in *GJB2* could influence protein function additively.

### *Analyses of* de novo *variants detected in the patient’s exome*

As we noticed that the c.262G pathogenic variant was *de novo*, we examined the *de novo* variants in the patient’s exome. It showed that there were approximately 47,000 variants, of which 143 variants were *de novo* (0.34%, 143/47,000). Among these 47,000 variants, 74 (0.016%, 74/47,000) were predicted to be pathogenic or likely pathogenic. Remarkably, 23 *de novo* variants were predicted to be pathogenic or likely pathogenic, including 21 heterozygous and two homozygous variants. The *de novo* adverse variants accounted for approximately one-third (23/74) of all pathogenic or likely pathogenic variants ( Table 2). Compared with the frequency of *de novo* variants in total being only 0.34%, the frequency of *de novo* adverse variants in all *de novo* variants reached above 16% (23/143) which is surprisingly high. The 23 *de novo* adverse variants were distributed in 19 different genetic areas, including 12 known genetic regions, two unclassified gene zones (LOC100509263 and LOC81691) and five other chromosome domains without defined roles ( Table 2). Except for the *de novo GJB2* c.262G>C variant, the other adverse variants have not yet been reported to be NSHL-causing.

Notably, eight copy number variations (CNVs) - nearly half of the infant’s 17 total adverse CNVs - were *de novo.* The *de novo* pathogenic CNVs accounted for 42% (8/19) of the total *de novo* adverse mutated genes ( Table 2); however, the minor allele frequencies of these CNVs were below 0.05 in the Human Gene Mutation Database and our in-house database (Joy Oriental Co., Table 2). Of these CNVs, six lacked the relevant information about their function, except the exon deletion in Rho Guanine Nucleotide Exchange Factor 5 ( *ARHGEF5*, exon 2-12, 13,006 bp) and Opsin 1, Medium Wave Sensitive 2 ( *OPN1MW2*, exon 1-6, 13,365 bp). The *ARHGEF5* and *OPN1MW2* gene products are crucial proteins that transduce external environmental cues into cellular signals across the cell membrane. Indeed, most CNVs do not encode important genes related to development and are thought to be subjected to adaptation to different environments. ^14^ Here, these recurrent *de novo* pathogenic CNVs in the patient remind us about the environmental influence on genetic components.

In total, four missense, four frameshift, three noncoding, two splice-site, one in-frame deletion and one stop gain variant which were predicted to be pathogenic/likely pathogenic, were *de novo* ( Table 2). Interestingly two heterozygous pathogenic variants in mitogen-activated protein kinase 8 interacting kinases 1 and 2 ( *MNK1* and *MNK2*) were *de novo*: one *MNK1* noncoding variant c.679 C>T in chromosome 11 (exon 12, MAF = 0.000046) and the other *MNK2* missense variant c.1816 G>A in chromosome 22 (exon 6, MAF unknown). Both MNK1 and MNK2 are serine/threonine kinases from the Ca ^2+^/calmodulin-dependent kinase family and take part in initiating mRNA translation in response to MAPK signaling, accordingly playing important roles concerning environmental stress and cytokines. ^15^ Also, four *de novo* pathogenic variants, including one homozygous missense variant c.11801C>T (MAF = 0.016), accumulated in the cell surface-associated Mucin 4 gene ( *MUC4*). Mucins are integral membrane glycoproteins on the cell surface. As the major constituents of mucus, mucins protect epithelial cells from outward stimuli. Additionally, two *de novo* heterozygous pathogenic variants c.1162-4(IVS9) insG (MAF unknown) and c.1162-5(IVS9) A>T (MAF = 0.000008) were detected in the Rad1-like checkpoint DNA exonuclease gene ( *RAD21*). The *RAD21* gene encodes the major cohesion subunit, known as the component of a heterotrimeric cell cycle checkpoint complex, regulating the segregation of sister chromatids in cell cycle progression and connecting inducible gene expression in response to diverse stimuli. ^16^ It is assumed that the proband’s genes with *de novo* pathogenic variants, including the disease-causing *GJB2* c.262G>C (p.A88P), were the key participants immediately linking the external stimuli and cellular signals. Therefore, we think that the causes of all these *de novo* adverse variants in the affected infant might.be directly linked to the fetal/maternal environmental factors.

## Discussion

This study examined the clinical/cytological characteristics and the compound heterozygous *GJB2* variants at c.79G>A, c.341A>G and c.262G>C in a Chinese family with a rare sporadic case of NSHL. In a previous cell-based functional assay, Zhang *et al*. demonstrated that the c.262G>T variant affected the intercellular exchange of larger molecules but left the ionic permeability intact, thus altering the kinetics of gap junction-mediated intercellular signaling and disrupting normal cochlear function. ^17^ Our study showed that the c.262G>T variant was expressed weakly and failed to regularly locate in the cell membrane, consequently reducing the formation of cell gap junctions. The *GJB2* p.[V27I; E114G] variant may also additively impair GJB2 function, as the channel activities of homozygous p.[V27I; E114G] CX26 gap junctions has been previously shown to be reduced. ^11^ Thus, it seems reasonable that carriers of the simple heterozygous c.262G>C could be asymptomatic, ^3^ while carriers of c.262G>C in compound heterozygosity along with any other deafness-related variants such as c.235delC, p.V27I, etc. could experience HL, like in our study and a previous NSHL case. ^3^ Hence, the pathogenic effects of these *GJB2* variants could be additive.

It should be noted that the penetrance of the *GJB2* c.262G>T seemed to be undetermined in the two previous NSHL cases: the heterozygous p.A88S in the Austrian patient with NSHL and his asymptomatic mother carrier ^2^; and the heterozygous p.A88V in the Japanese girl with severe keratitis-ichthyosis-deafness syndrome and her healthy parents. ^6^ Both studies reported no other *GJB2* variants except the heterozygous c.262G. In fact, only the candidate *GJB2* genetic region was sequenced in their studies, so any other genetic disease-causing variants in the patients’ genome are still unknown. Therefore, for an accurate variant interpretation and improved clinical care, we propose that more comprehensive details about the related variants, such as variant domain, effect, and reciprocal interaction, should be investigated.

In the current study, it is intriguing that there was a very high frequency of *de novo* adverse variants in the proband’s exome and that most *de novo* variants are in the genetic regions characterized as environment-sensitive. Several notable results were found. First, the gene in which the *de novo* NSHL-causing *GJB2* c.262G>C is located is immediately responsive to the surrounding changes. The gene product GJB2 is essential for gap channels, which allows the exchange of small substances including nutrients, metabolites, ions and second messengers, and regulates signaling pathways in intracellular communication. ^1^^,^^17^ Second, variants in *MNK1* and *MNK2 -* two downstream MAPK signaling effectors located in different chromosomes - were also *de novo.* Both MNKs are involved in guiding cellular responses to a diverse array of stimuli, such as mitogens, osmotic stress, heat shock and proinflammation. ^15^^,^^18^ Third, the *de novo* pathogenic variants were aggregated in the *MUC4* gene region. Mucins are integral membrane glycoproteins on the cell surface, covering epithelial surfaces such as those in the trachea, colon and cervix, and exert anti-adhesive effects on cell-cell and cell-extracellular matrix interactions. ^19^ Fourth, there were two *de novo* pathogenic variants in the *RAD21* gene. RAD21 participates in repairing DNA double-strand breaks and chromatid cohesion and can be affected by various agents, including ionizing radiation, topoisomerase inhibitors, cycloheximide, proteasome inhibitors, cytokines agents and inflammatory stimuli. ^16^^,^^20^ Finally, there was a very high incidence of *de novo* pathogenic CNVs which have quite low MAFs. CNVs are often enriched in genes related to sensory perception of the external environment (e.g., smell, sight, and taste), neurodevelopmental processes, and response to chemical stimuli, immunity and other processes. ^14^ Therefore, we wonder whether there might have been any direct external stimuli to trigger the fetal adaptive responses for the occurrence of such a considerable amount of *de novo* pathogenic variants, which thus led to the disease.

It should be noted that the sporadic congenital NSHL in the family that we studied here was rare and limited; more research in similar birth defect cases is needed to confirm the role of environmental factors in transformation of *de novo* genetic variants in the fetus/offspring. Also, sequencing errors remain one of the main obstacles in the identification of causative genetic variants and/or mutations. However, in the research for the genetic basis of severe childhood-onset disorders, it is not scarce that the *de novo* genetic variants could be pathogenic, for example, the typical cause for childhood cardiomyopatheis was most commonly *de novo* mutations, although the background for such variants is poorly characterized. ^21^

In summary, by whole-exome sequencing, we examined overall genetic variants, especially the compound heterozygous *GJB2* variants and the high frequency of *de novo* pathogenic variants in a Chinese family with a rare sporadic case of NSHL. Though the reported case here is limited, we think the detailed full picture of genetic variants could improve our interpretation of the HL-associated genetic variants. In order to further advance our understanding of disease biology in birth defects, further research on environmental causes for *de novo* pathogenic variants may be needed.

## Data availability

### Underlying data

Open Science Framework: Whole-exome sequencing of *de novo* genetic variants in a Chinese family with a sporadic case of congenital nonsyndromic hearing loss. https://doi.org/10.17605/OSF.IO/DS7TW. ^22^

This project contains the following underlying data:

- The immunoblotting of the p.A88P Cx26 mutants in cells. (mut*.tif)
- The immunoblotting of the wild type Cx26 in cells. (WT*.tif)
- The data comparison of the exome DNA sequences of the family members. (*.xlsx)

NCBI Gene: Exome sequencing of a Chinese family with a sporadic congenital NSHL. Accession number **PRJNA688744**.

Data are available under the terms of the Creative Commons Attribution 4.0 International license (CC-BY 4.0).

## Ethical approval

The data were de-identified as sufficiently as possible and data sharing was approved by the Research Ethics Committee of Sichuan Provincial People’s Hospital, School of Medicine, UESTC (approval number 2019-065).
