# Supplementary material for: Whole-exome sequencing of de novo genetic variants in a Chinese family with a sporadic case of congenital nonsyndromic hearing loss
Source: F1000Res. 2021 Aug 24;10:61. Originally published 2021 Feb 2. [Version 2] doi: 10.12688/f1000research.27739.2 (PMC8422347; doi:10.12688/f1000research.27739.2)
Supplement: Peer review history

# Peer review: Reviewer response for version 2

**DOI:** 10.5256/f1000research.57079.r92750

**Published:** 2021-09-06

**Authors:** Yuande Tan

**Affiliations:** 1 Institute of Personalized Medicine, PENN State University, Hershey College of Medicine, Hershey, PA, USA

All my concerns are addressed. All minor points are clear, and errors or typos are corrected. For these questions above are clearly answered.

Is the work clearly and accurately presented and does it cite the current literature?

Partly

If applicable, is the statistical analysis and its interpretation appropriate?

Partly

Are all the source data underlying the results available to ensure full reproducibility?

Partly

Is the study design appropriate and is the work technically sound?

Partly

Are the conclusions drawn adequately supported by the results?

Partly

Are sufficient details of methods and analysis provided to allow replication by others?

Partly

Reviewer Expertise:

NA

I confirm that I have read this submission and believe that I have an appropriate level of expertise to confirm that it is of an acceptable scientific standard.

# Peer review: Reviewer response for version 1

**DOI:** 10.5256/f1000research.30672.r82018

**Published:** 2021-04-15

**Authors:** Yuande Tan

**Affiliations:** 1 Institute of Personalized Medicine, PENN State University, Hershey College of Medicine, Hershey, PA, USA

This paper displays a variant profile of a Chinese family with a case of congenital nonsyndromic hearing loss using the whole-exome sequencing data. Authors attempted to utilize heterozygote of a de nova variant (c.262G>C) in gene GJB2 to explain case of nonsyndromic hearing loss occurring in an offspring individual. However, I have several major concerns to be addressed:  

1. The results show among the total~47,000 variants, 143 were de novo occurring in the patient. According to definition of de nova from Wikipedia, a de nova variant is a variant occurring in offspring individuals, not in parents, that is, de nova variants results from mutation occurring in sperm or germ cells. However, 143/47000(0.304%) is too high. Since there is one offspring individual in this study, so, it is very difficult to determine whether these de nova variants are due to noise or true mutation. In addition,47,000 variants detected in the patient are also too many and most of these so-called variants may be noisy or result from sequencing error. Authors should discuss this issue.
2. If it is known that nonsyndromic hearing loss is due to mutation in gene GJB2, it is unnecessary to do the whole exom sequencing, that is to say, the whole variant profiles of parents and daughter (proband) do not provide useful information for interpreting the nonsyndromic hearing loss. The de nova variants listed in Table 2 also do not make sense because they are not used to explain occurrence of the nonsyndromic hearing loss.
3. How heterozygous c.262G>C missense variants works for occurrence of the nonsyndromic hearing loss is not clear. Since the farther and mother have homozygote c.262GG and have no nonsyndromic hearing loss and heterozygous c.262G>C  variant was also found in healthy individuals, the proband with heterozygous c.262G>C  variant at this position in gene GJB2 should be normal hearing. However, the proband had heterozygous variants c.79G>A and c.341A>G in gene GJB2 derived from her mother but her mother is normal hearing. Therefore, there may be interaction (dominant epistasis or receive epistasis) between c.262G>C and c.79G>A or c.341A>G. Author should discuss this possible heredity mechanism.

Minor points:

1. On page 3, simple, monogenetic should be simple and monogenetic.
2. On page 3, “another was the c.263C>A (p.A88E) variant” should be “another case was the c.263C>A (p.A88E) variant”.
3. On page 3,   “c.235delC5 ; and another was the c.263C>T (p.A88V) variant” should be   “c.235delC5  and the other variant was the c.263C>T (p.A88V)”.
4. On page 3, “several clinical studies have found the p.[V27I; E114G] haplotype to be a risk factor” should be “several clinical studies have found that the p.[V27I; E114G] haplotype is a risk factor”.
5. On page 3, “Written informed consent was obtained” should be “An informed consent was obtained”.
6. On page 4, what is “size distribution and concentration”?
7. On page 5, “the affected infant is considered to be a sporadic case of NSHL”. I don’t think it is a sporadic case because you just found one case and you cannot determine it is a sporadic case or a pedigree case or a family case. 
8. On page 5, what is “large deletions”?
9. On page 5, “The exome sequences revealed no known NSHL-causing variants” should be “The exome sequences revealed unknown NSHL-causing variants”.
10. On page 5, “whereas her parents were wild type” should be “whereas her parents were normal type”. I have never seen “wild type” in human.
11. Legend Figure 1 is unclear. It does not state what is Figure 1A.

Is the work clearly and accurately presented and does it cite the current literature?

Partly

If applicable, is the statistical analysis and its interpretation appropriate?

Partly

Are all the source data underlying the results available to ensure full reproducibility?

Partly

Is the study design appropriate and is the work technically sound?

Partly

Are the conclusions drawn adequately supported by the results?

Partly

Are sufficient details of methods and analysis provided to allow replication by others?

Partly

Reviewer Expertise:

NA

I confirm that I have read this submission and believe that I have an appropriate level of expertise to confirm that it is of an acceptable scientific standard, however I have significant reservations, as outlined above.

# Response

**Published:** 2021-05-21

**Authors:** Sonia Liao

**Affiliations:** UESTC, China

1.    The results show among the total~47,000 variants, 143 were de novo occurring in the patient. According to definition of de nova from Wikipedia, a de nova variant is a variant occurring in offspring individuals, not in parents, that is, de nova variants results from mutation occurring in sperm or germ cells. However, 143/47000(0.304%) is too high. Since there is one offspring individual in this study, so, it is very difficult to determine whether these de nova variants are due to noise or true mutation. In addition,47,000 variants detected in the patient are also too many and most of these so-called variants may be noisy or result from sequencing error. Authors should discuss this issue.

  Our answer: Thanks. We discussed that “sequencing errors remain one of the main obstacles in the identification of causative genetic variants and/or mutations” in the new version, please see the 4th paragraph of the discussion section. 

 We may need to mention here, the genetic counseling center in our hospital, one of the earliest clinic genetic research groups in China, offers regular exome sequencing to identify and study disease-related genetic mutations for patients. For the quality of our exome sequencing in the current work, please refer to the chapter of Whole-exome and mitochondrial DNA sequencing in the materials and methods. And also, all sequence information is open at NCBI Gene: Exome sequencing of a Chinese family with a sporadic congenital NSHL. Accession number PRJNA688744. 

 2.    If it is known that nonsyndromic hearing loss is due to mutation in gene GJB2, it is unnecessary to do the whole exom sequencing, that is to say, the whole variant profiles of parents and daughter (proband) do not provide useful information for interpreting the nonsyndromic hearing loss. The de nova variants listed in Table 2 also do not make sense because they are not used to explain occurrence of the nonsyndromic hearing loss.

  Our answer: Thanks. As we mentioned in the 1st paragraph of the introduction: “GJB 2 have been shown to be the leading genetic cause of NSHL (OMIM: 121011). GJB2-related autosomal recessive deafness can explain approximately 50% of congenital autosomal recessive deafness.” In fact, pathogenic variants were found in 49 genes according to GeneReviews of NSHL (https://www.ncbi.nlm.nih.gov/books/NBK1272/). In the current case of an NSHL newborn, we think that it is necessary to carry out the whole-exome sequencing to search for the genetic causes of the disease.

 In the new version, we added that “the pathogenic variants of non-syndromic sensorineural hearing loss (NSHL) (OMIM: 121011) were found in 49 genes (https://www.ncbi.nlm.nih.gov/books/NBK1272/)” in the 1st paragraph of the introduction, in order to emphasize that GJB2 variants are not the only genetic causes of NSHL, and also, we pointed out that “We carried out whole-exome sequencing, assessed the cytological/clinical characteristics of the genetic variants, specifically in the GJB2 genetic variants, and evaluated the possible cause of de novo pathogenic variants in the patient’s exome.” in the last paragraph of the introduction.

 3.    How heterozygous c.262G>C missense variants works for occurrence of the nonsyndromic hearing loss is not clear. Since the farther and mother have homozygote c.262GG and have no nonsyndromic hearing loss and heterozygous c.262G>C  variant was also found in healthy individuals, the proband with heterozygous c.262G>C  variant at this position in gene GJB2 should be normal hearing. However, the proband had heterozygous variants c.79G>A and c.341A>G in gene GJB2 derived from her mother but her mother is normal hearing. Therefore, there may be interaction (dominant epistasis or receive epistasis) between c.262G>C and c.79G>A or c.341A>G. Author should discuss this possible heredity mechanism.

  Our answer: Thanks. According to the definition of National Human Genome Research Insititute, epistasis refers to a circumstance where the expression of one gene is affected by the expression of one or more independently inherited genes (https://www.genome.gov/genetics-glossary/Epistasis).  In the current study, both c.262G>C and the haplotype of c.79G>A and c.341A>G are in the exon2 of GJB2 gene, and encode amino acids which were tightly connected in this protein (for details please see the figure2). We do not think that it should be necessary to discuss about the independent heredity mechanism of these variants in the same gene. Additionally, in our study, we noticed the insufficient GJB2 function because of these variants, while we have not seen any report about the epistasis in this exact situation. 

 Minor points:

 1.    On page 3, simple, monogenetic should be simple and monogenetic.

   Our answer: According to the suggestion, we modified the text.

 2.    On page 3, “another was the c.263C>A (p.A88E) variant” should be “another case was the c.263C>A (p.A88E) variant”.

    Our answer: The “case” was added in the context of the whole sentence.

 3.    On page 3,   “c.235delC5 ; and another was the c.263C>T (p.A88V) variant” should be   “c.235delC5  and the other variant was the c.263C>T (p.A88V)”.

     Our answer: Thanks. This sentence might be confusing due to the typesetting errors? “c.235delC5” was not correct here. Here, we listed that researchers have reported 3 p.A88 coding variants at the 263rd nucleotide: (1) c.263C>G (p.A88G) [reference4], (2) c.263C>A (p.A88E) in compound heterozygosity with c.235delC [reference5], (3) c.263C>T (p.A88V) [reference5]. 

 Since there were totally 3 reported cases, the sentence we presented them like: “there are three pens, one is red, another is black, and another is green”

 4.    On page 3, “several clinical studies have found the p.[V27I; E114G] haplotype to be a risk factor” should be “several clinical studies have found that the p.[V27I; E114G] haplotype is a risk factor”.

    Our answer: We modified the text according to the suggestion.

 5.    On page 3, “Written informed consent was obtained” should be “An informed consent was obtained”.

     Our answer: Thanks. We found that “written informed consent” without any prefix ( “a” or “the”) was used in many research articles,  and listed below 2 of these examples from the Pubmed for your reference.

 “Consent for publication Written informed consent was obtained from the patient for the publication of this report and any accompanying images.”

 Reich M, Cakir B, Cvetkoski S, Lang SJ, Stahl A, Ness T, Agostini H, Lange C. Acute unilateral maculopathy associated with adult onset of hand, foot and mouth disease: a case report. BMC Ophthalmol. 2019; 19(1):104. doi: 10.1186/s12886-019-1111-4.

 “Informed consent Written informed consent was obtained from the patient for publication of this case report.”

 Lieberman A, Curtis L.Severe Adverse Reactions Following Ketoconazole, Fluconazole, and Environmental Exposures: A Case Report. Drug Saf Case Rep. 2018 18;5(1):18. doi: 10.1007/s40800-018-0083-2.

 And also, there are the same sentence examples at the websites of English sources like: https://www.lawinsider.com/dictionary/informed-written-consent and https://ludwig.guru/s/written+informed+consent+was+obtained+from+all+subjects.

 6.    On page 4, what is “size distribution and concentration”?

  Our answer: Here the “size distribution and concentration” belong to the qPCR of the enriched DNA fragments; we changed it into “the size distribution and concentration of these DNA fragments were examined…” Please see the revised sentence in the new version. 

 7.    On page 5, “the affected infant is considered to be a sporadic case of NSHL”. I don’t think it is a sporadic case because you just found one case and you cannot determine it is a sporadic case or a pedigree case or a family case. 

   Our answer: Commonly for patients with birth defect, we investigated their family history for any inheritance diseases; if we got no report of such case in their family, we would consider that the new case could be isolated and sporadic. 

 We think that, to an individual family with birth defect, this kind of considerations and the searching for genetic causes of the disease could be useful, especially when these families want to prevent and intervene more disease happening. 

 We believe that current case report about the de novo pathogenic variants could provide new and useful information that lead to further and vital research. 

 On page 5, what is “large deletions”?

  Our answer: According to the National Human Genome Research Institute: “deletion can be small, involving a single missing DNA base pair, or large, involving a piece of a chromosome”. Large deletions in genomic DNA have been reported to associate with many diseases. For an example: 

 Yu CE, Dawson G, Munson J, D'Souza I, Osterling J, Estes A, Leutenegger AL, Flodman P, Smith M, Raskind WH, Spence MA, McMahon W, Wijsman EM, Schellenberg GD. Presence of large deletions in kindreds with autism. Am J Hum Genet. 2002; 1(1):100-15. doi: 10.1086/341291

 In our study, because large deletions in mitochondrial DNA have been reported to associate with hearing loss [some related references were listed below], we stated our sequencing result as that “the mitochondrial sequencing showed no NSHL-causing variants or large deletions.”

 Souied EH, Salès MJ, Soubrane G, Coscas G, Bigorie B, Kaplan J, Munnich A, Rötig A. Macular dystrophy, diabetes, and deafness associated with a large mitochondrial DNA deletion. Am J Ophthalmol. 1998; 125(1):100-3. doi: 10.1016/s0002-9394(99)80243-8.

 Yin S, Yu Z, Sockalingam R, Bance M, Sun G, Wang. The role of mitochondrial DNA large deletion for the development of presbycusis in Fischer 344 rats. J. Neurobiol Dis. 2007; 27(3):370-7. doi: 10.1016/j.nbd.2007.06.006.

 8.    On page 5, “The exome sequences revealed no known NSHL-causing variants” should be “The exome sequences revealed unknown NSHL-causing variants”.

 Our answer:  Here we want to say that “The exome sequences did not revealed any known NSHL-causing variants”, NOT about any unknown NSHL-causing variants.

   

 9.    On page 5, “whereas her parents were wild type” should be “whereas her parents were normal type”. I have never seen “wild type” in human.

 Our answer:  Thanks. We searched “wild type in human” in the Pubmed and listed some results here for your reference:

 Serebryany E, King JA. Wild-type human γD-crystallin promotes aggregation of its oxidation-mimicking, misfolding-prone W42Q mutant. J Biol Chem. 2015; 290(18):11491-503. doi: 10.1074/jbc.M114.621581.

 Graffmo KS, Forsberg K, Bergh J, Birve A, Zetterström P, Andersen PM, Marklund SL, Brännström T. Expression of wild-type human superoxide dismutase-1 in mice causes amyotrophic lateral sclerosis. Hum Mol Genet. 2013; 22(1):51-60. doi: 10.1093/hmg/dds399. 

 Friedman PN, Kern SE, Vogelstein B, Prives C. Wild-type, but not mutant, human p53 proteins inhibit the replication activities of simian virus 40 large tumor antigen. Proc Natl Acad Sci U S A. 1990 Dec;87(23):9275-9. doi: 10.1073/pnas.87.23.9275.

 10.    Legend Figure 1 is unclear. It does not state what is Figure 1A.

 Our answer:  Thanks. We made it clear now that figure1A shows the pedigree of the family in the new version.

# Peer review: Reviewer response for version 1

**DOI:** 10.5256/f1000research.30672.r82648

**Published:** 2021-04-08

**Authors:** Kun Zhang

**Affiliations:** 1 Department of Genetics, School of Bioscience and Technology, Chengdu Medical College, Chengdu, China

The authors report an interesting observation describing a patient with non-syndromic hearing loss caused by a de novo heterozygous variant of GJB2 c.262G>C. The authors identified the variant by whole exome sequencing and provided biological evidence of the variant by in vitro analysis.

 Here, several concerns were raised:

 The number of de novo variants (23) seemed very high. De novo genetic variants are recently suggested to be important in human disease, one investigation of severe childhood cardiomyopathy identified a de novo variant in 46% of children with a pathogenic variant (Vasilescu et al., Genetics basis of severe childhood onset cardiomyopathies. Journal of the American College of Cardiology 2018), yet the overall frequency of de novo variants is largely unknown. Should the authors discuss more about the issue? It should be noted that one case here is limited.

Is the work clearly and accurately presented and does it cite the current literature?

Yes

If applicable, is the statistical analysis and its interpretation appropriate?

Yes

Are all the source data underlying the results available to ensure full reproducibility?

Yes

Is the study design appropriate and is the work technically sound?

Yes

Are the conclusions drawn adequately supported by the results?

Yes

Are sufficient details of methods and analysis provided to allow replication by others?

Yes

Reviewer Expertise:

Medical Genetics

I confirm that I have read this submission and believe that I have an appropriate level of expertise to confirm that it is of an acceptable scientific standard.

# Response

**Published:** 2021-05-21

**Authors:** Sonia Liao

**Affiliations:** UESTC, China

Thanks a lot. We presented ****the suggested reference in the new version, please see the 4 ^th^ paragraph of the discussion section..
